# Treatment of Diabetes

**Published:** 1909-09

**Authors:** 


### ABSTRACTS.

### TREATMENT OF DIABETES.

Dr. Manfred Fraenkel of Berlin, (*Med. Klink,* 1905, Nos. 55-56,) presented a new theory of the pathogenesis of diabetes, based on the idea that normally the transformation of glycogen into sugar is due to a ferment which arises from the decomposition of red blood corpuscles. This ferment is produced more rapidly when there are circulatory disturbances,—until a point is reached when the quantity of sugar created no longer can be utilised, supersaturates the blood, and is excreted in the urine.

A condition for normal utilisation of sugar is a normally functioning vasomotor system, with its center on the floor of the rhomboid fossa, a normal pancreas. The trophic factors are of no small importance. A dominating position over the entire vasomotor system of the liver must be ascribed to the vagus. In this connection the relationship between diabetes and tuberculosis is of much interest. Bernard found sugar-forming fibers of the vagus, so that any injury to the former must also strike the latter. This seems to explain the secondary occurrence of tuberculosis in diabetes.

Fraenkel then points out the possibility of influencing the vagus by means of eserine. He considers the activity and pathology of the pancreas and the pathology of diabetes in general, and emphasises that changes in the pancreas present themselves as interstitial degeneration, especially of Langerhan's cells. These, too, have their cause in the circulatory disturbances. In diabetes all other organs always show signs of extensive hyperemia. The final link in his chain of reasoning is the significance of arteriosclerosis. He cites Noorden and Croner in support of the connection between it and diabetes. Arteriosclerosis is primarily the expression of circulatory disturbances, and according to the location of the vascular injury one subject is exempt from diabetes, while another succumbs thereto when the arteriosclerosis establishes itself in the hepatic vessels.

He therefore combined eserine with the modified salts of Trunecek's serum (antisclerosin), the tablets being given the name of "diabeteserin," and used the same with very good results in 22 of 29 cases, which he demonstrates by several histories.

This preliminary publication has found confirmation in the reports of Markbreiter (*Wien. med. Presse,* 1906, No. 36); Assmann (*Medico,* 1906, No. 22); Huber (*Zentralblatt f. d. ges. Therapie,* 1906, No. 9); and Friedmann (*Oesterr. Aerzte-Zeitung,* 1906, No. 12).

Diabeteserin is furnished in two forms. Diabeteserin No. 1 contains the blood salts with 0,0003 gm. physosligmine salicylate per tablet. Diabeteserin No. 2 contains, in addition, 0,00005 gm. atropine per tablet. No. 2 is ordered is cases with obstinate constipation, colicky pains, or pronounced obesity, when No. 1 fails to give the desired results.

The dosage is one or two tablets thrice daily for from two to four weeks. The diet, of course, must be adapted to the requirements of the individual.

---

